# Liver-specific knockout of B cell lymphoma 6 suppresses progression of non-alcoholic steatohepatitis in mice

**DOI:** 10.1038/s41598-020-66539-z

**Published:** 2020-06-16

**Authors:** Hiromi Chikada, Kinuyo Ida, Yuji Nishikawa, Yutaka Inagaki, Akihide Kamiya

**Affiliations:** 1 Department of Molecular Life Sciences, Tokai University School of Medicine, 143 Shimokasuya, Isehara, Kanagawa Japan 259-1193; 2 Center for Matrix Biology and Medicine, Tokai University School of Medicine, 143 Shimokasuya, Isehara, Kanagawa Japan 259-1193; 3 Department of Innovative Medical Science, Tokai University School of Medicine, 143 Shimokasuya, Isehara, Kanagawa Japan 259-1193; 4 Department of Pathology, Asahikawa Medical University, Asahikawa, Hokkaido, Japan

**Keywords:** Non-alcoholic fatty liver disease, Non-alcoholic steatohepatitis

## Abstract

The prevalence of non-alcoholic steatohepatitis (NASH) rapidly increases with metabolic disorders such as dyslipidaemia, high blood pressure, and hyperglycaemia. B cell lymphoma 6 (Bcl6), a transcriptional repressor, is essential for the formation of germinal centre B cells. In this study, we analysed the role of Bcl6 in NASH progression-associated pathological changes, such as hepatic lipid accumulation, liver fibrosis, and hepatocarcinogenesis. The roles of Bcl6 in NASH were analysed using liver-specific Bcl6 knockout (Bcl6-LKO) and control wild-type (WT) mice. The murine NASH model was established by feeding the mice with choline-deficient, L-amino-acid-defined, high-fat diet (CDAHFD). Feeding the WT mice with CDAHFD for 7 weeks induced the formation of histopathological features resembling human NASH, such as hepatic lipid accumulation, hepatocellular injury, and fibrosis. These histopathological changes were significantly attenuated in Bcl6-LKO mice. Additionally, feeding the male WT mice with CDAHFD for 38 weeks induced the formation of liver tumours, which was suppressed in Bcl6-LKO mice. These findings indicate that Bcl6 is involved in the progression of NASH and NASH-derived tumours.

## Introduction

Globally, hepatocellular carcinoma is associated with high morbidity and mortality rates. The major etiological factor for hepatocellular carcinomas is chronic hepatitis, which is induced by infections from hepatitis B virus (HBV) and hepatitis C virus (HCV). The non-viral etiological factors for hepatocellular carcinoma are excessive alcohol and lipid intake. The symptoms of hepatitis and hepatocellular carcinomas induced by HBV and HCV can be controlled by antiviral drugs, such as nucleic acid analogue preparations for HBV and direct-acting antivirals for HCV^1^.

In addition to the incidences of visceral fat-type obesity, the incidences of metabolic disorders, such as high blood pressure, hyperglycaemia, and dyslipidaemia are rapidly increasing due to unhealthy lifestyle habits, such as the consumption of high-fat diet and lack of exercise. Non-alcoholic fatty liver disease (NAFLD), a metabolic syndrome, shows abnormal lipid accumulation in the liver. Many patients with NAFLD do not exhibit major pathological symptoms, except simple fatty livers. However, NAFLD may progress to hepatitis and liver cirrhosis in some cases and liver tumours in severe cases, which could be lethal. The progression of non-alcoholic steatohepatitis (NASH) has classically been explained by two-hit hypothesis^2^. Steatohepatitis is induced by abnormal accumulation of lipids in the liver, an upstream pathological change, followed by increased oxidative stress and enhanced secretion of inflammatory cytokines. The prolonged inflammatory condition promotes the progression from liver fibrosis to cirrhosis and hepatocellular carcinomas. There are multiple risk factors reported for various NASH pathologies, which has led to the proposal of parallel hits hypothesis for the progression of NASH^3^. Recently, there has been increased incidence of serious liver diseases, such as liver tumours caused by NASH. Thus, there is a need to elucidate the mechanism underlying NASH progression to liver tumours to devise preventive and therapeutic strategies^4,5^.

B cell lymphoma 6 (Bcl6), an important transcription repressor in the immune system, is essential for germinal centre B cell formation. The mice that lack Bcl6 in the whole-body exhibit myocarditis^6,7^^,^. A chromosomal translocation at the Bcl6 locus is reported to cause lymphoma. In lymphoma, Bcl6 is reported to be an oncogene^8^. Previous studies have revealed that Bcl6 is involved in lipid metabolism. A study using whole-body Bcl6-deficient mice reported that Bcl6 regulates fatty liver synthesis in liver^9^. In addition, Bcl6 regulates insulin sensitivity and distribution of body fat using mice with Bcl6 deficiency in fat tissue^10^. However, the functions of Bcl6 in the mature liver have not been evaluated because most whole-body Bcl6-deficient mice die within 5-9 weeks of birth^7^. Thus, liver-specific Bcl6 knockout mice (Bcl6-LKO mice) are an appropriate model to analyse the function of Bcl6 in the adult liver. We reported that Bcl6 regulates the expression of cytochrome P450 metabolic enzymes in the liver using Bcl6-LKO mice^11^. Moreover, Sommars *et al*. recently reported that Bcl6 expression in the liver was important for the regulation of β-oxidation^12^. These findings indicate that Bcl6 is involved in several liver metabolism such as lipid metabolism. However, the role of Bcl6 in the progression of NASH or NASH-derived tumours has not been studied.

In this study, we analysed the potential roles of Bcl6 in NASH progression and NASH-induced hepatocarcinogenesis in mice fed with choline-deficient, L-amino-acid-defined, high-fat diet (CDAHFD). This diet markedly increased the rates of hepatic injury, hepatic lipid accumulation, and hepatic fibrosis in wild-type (WT) mice. However, the Bcl6-LKO mice exhibited suppressed progression of NASH. Moreover, hepatocarcinogenesis induced by the consumption of CDAHFD for 38 weeks in male WT mice was significantly reduced in Bcl6-LKO mice. These results indicated that Bcl6 was involved in the progression of NASH and NASH-derived tumours.

## Results

### Serum profiles of Bcl6-LKO mice

**Figure 1:**
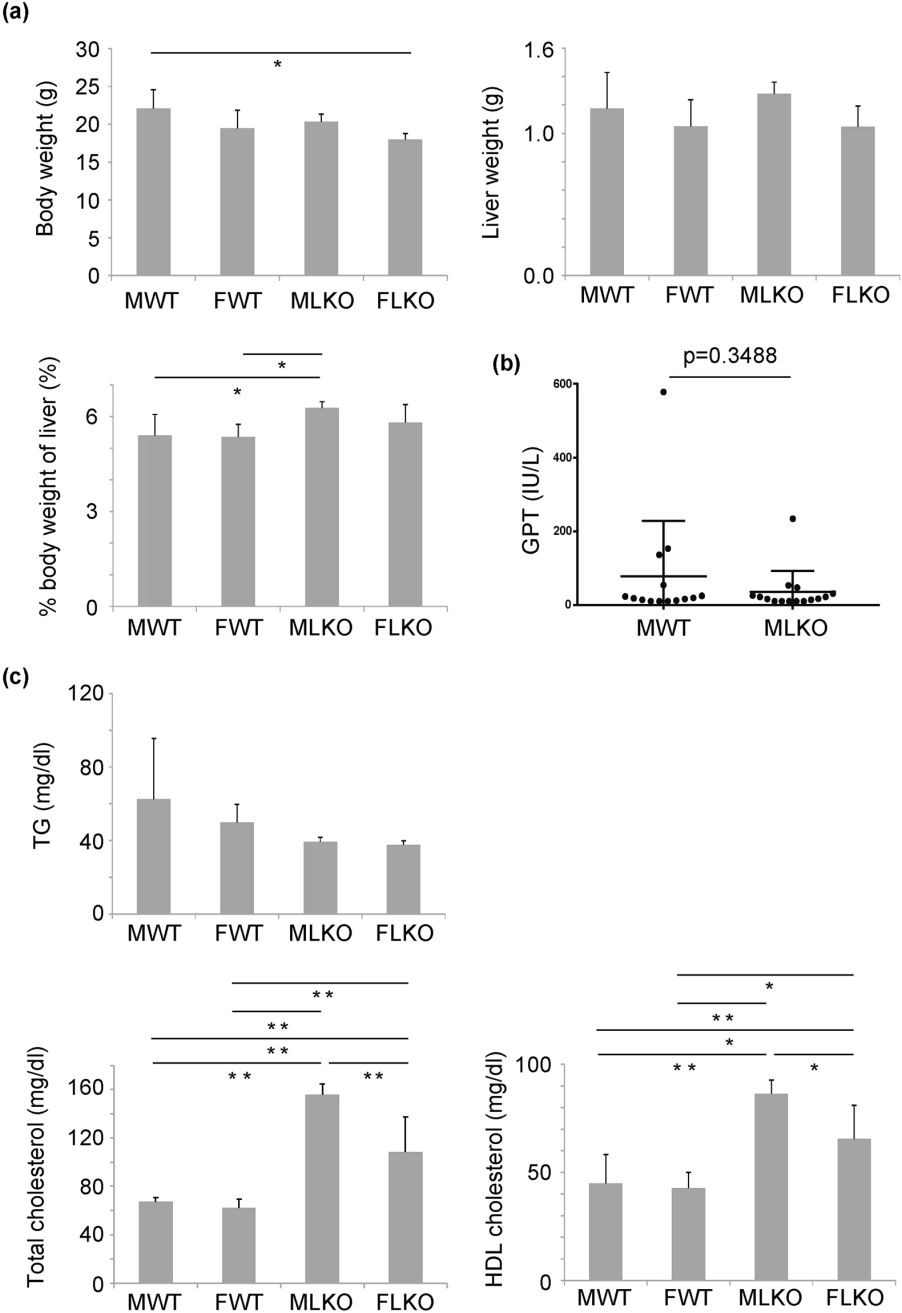
Analysis of the characteristics of liver-specific Bcl6 knockout (Bcl6-LKO) mice fed with a standard diet. (**a**) Body weight and liver weight of 10-week-old mice were measured. The percent weight of the liver relative to the body weight (% body weight of liver) was calculated. Results are represented as mean ± standard deviation (S.D.) (n = 6 for male wild-type mice, n = 5 for female wild-type mice, n = 6 for male Bcl6-LKO mice, n = 4 for female Bcl6-LKO mice). (**b**) The serum level of glutamic pyruvic transaminase (GPT) was used as an indicator of liver injury. (n = 14 for male wild-type feeding and fasting mice, n = 15 for male feeding and fasting Bcl6-LKO mice). (**c**) The serum levels of triglyceride, total cholesterol, and high-density lipoprotein (HDL) cholesterol. Results are represented as mean ± S.D. (n = 6 for male wild-type mice, n = 5 for female wild-type mice, n = 6 for male Bcl6-LKO mice, n = 4 for female Bcl6-LKO mice). *P < 0.05, **P < 0.01. MWT, male wild-type mouse samples; FWT, female wild-type mouse samples; MLKO, male Bcl6-LKO mouse samples; FLKO, female Bcl6-LKO mouse samples.

The Bcl6-LKO mice were generated by crossing Bcl6-floxed mice with albumin-Cre mice as described previously^11^. The body weight and liver weight, as well as the serum lipid components, of Bcl6-LKO mice were analysed. The body weight and liver weight of whole-body Bcl6 deficient mice were reported to be lower than those of WT mice^9^. The body weight of Bcl6-LKO mice aged 7–10 weeks was not significantly different from that of age-matched WT mice (Fig. 1a and Supplementary Fig. S1a) whereas the body weight of Bcl6-LKO mice aged 6 weeks was slightly lower than that of age-matched WT mice (Supplementary Fig. S1b, 6 age in weeks). Additionally, the liver weight of 10-week-old Bcl6-LKO mice was not significantly different from that of age-matched WT mice after standard diet feeding (Fig. 1a). The percent weight of liver relative to the body weight in Bcl6-LKO mice was slightly higher than that in WT mice. The levels of glutamic pyruvic transaminase (GPT), a hepatocytic injury marker, were similar between WT and Bcl6-LKO mice (Fig. 1b).

Next, we analysed the serum lipid component in Bcl6-LKO mice. The levels of serum triglyceride were not significantly different between WT and Bcl6-LKO mice. However, the serum total cholesterol and high-density lipoprotein (HDL) cholesterol levels in Bcl6-LKO mice were significantly higher than those in WT mice (Fig. 1c).

### Analysis of hepatic lipid metabolism in Bcl6-LKO mice

**Figure 2:**
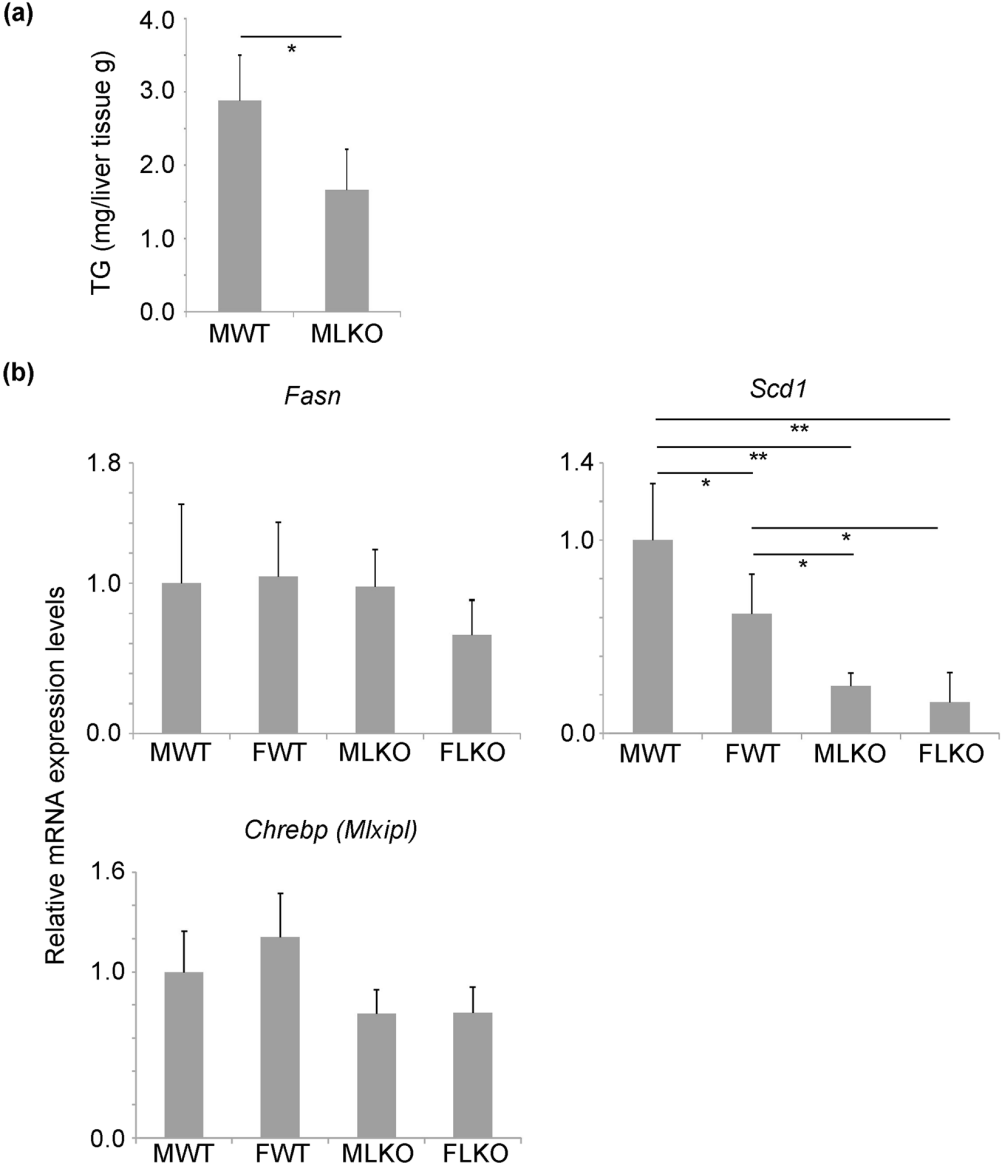
Changes in lipid metabolism in Bcl6-LKO mice (**a**) The liver triglyceride (TG) level was measured. Results are represented as mean ± standard deviation (S.D.) (n = 5). (**b**) The mRNA expression levels of *Fasn*, *Scd1*, and *Chrebp* in mice fed with standard diet were measured by quantitative real-time polymerase chain reaction. The *Hprt* gene was used as an internal control. The expression of genes in the liver of male wild-type mice was set to 1.0. Results are represented as mean ± S.D. (n = 6 for male wild-type mice, n = 5 for female wild-type mice, n = 6 for male Bcl6-LKO mice, n = 4 for female Bcl6-LKO mice). *P < 0.05, **P < 0.01 MWT, male wild-type mouse samples; FWT, female wild-type mouse samples; MLKO, male Bcl6-LKO mouse samples; FLKO, female Bcl6-LKO mouse samples.

Next, we analysed the hepatic triglyceride level in Bcl6-LKO mice. The hepatic levels of triglyceride in Bcl6-LKO mice were significantly suppressed, which was also reported in whole-body Bcl6 deficient mice^9^ (Fig. 2a). The expression levels of genes related to lipid metabolism were analysed by quantitative real-time polymerase chain reaction (qRT-PCR). The mRNA expression levels of fatty acid synthetase gene (*Fasn*) were similar between Bcl6-LKO and WT mice. The mRNA expression levels of stearoyl-CoA desaturase 1 gene (*Scd1*) in Bcl6-LKO mice were downregulated when compared with those in WT mice (Fig. 2b). Additionally, the mRNA expression levels of *Chrebp*, the transcription factor regulating the expression of *Fasn* and *Scd1*, were not significantly different between WT and Bcl6-LKO mice (Fig. 2b).

Recently, hepatocytic Bcl6 was reported to regulate the expression of genes related to β-oxidation^12^. In this study, we confirmed that the expression levels of β-oxidation-related genes, such as ATP-binding cassette sub-family D member 1 (*Abcd1*), acyl-coenzyme A amino acid N-acyltransferase 2 (*Acnat2*), acyl-CoA thioesterase 4 (*Acot 4*), hydroxyacyl-coenzyme A dehydrogenase (*Hadh*), acyl-CoA dehydrogenase very long chain (*Acadvl*), and uncoupling protein 2 (*Ucp 2*) were upregulated in the liver of Bcl6-LKO mice (Supplementary Fig. S2).

### Analysis of lipoprotein transport in Bcl6-LKO mice

**Figure 3:**
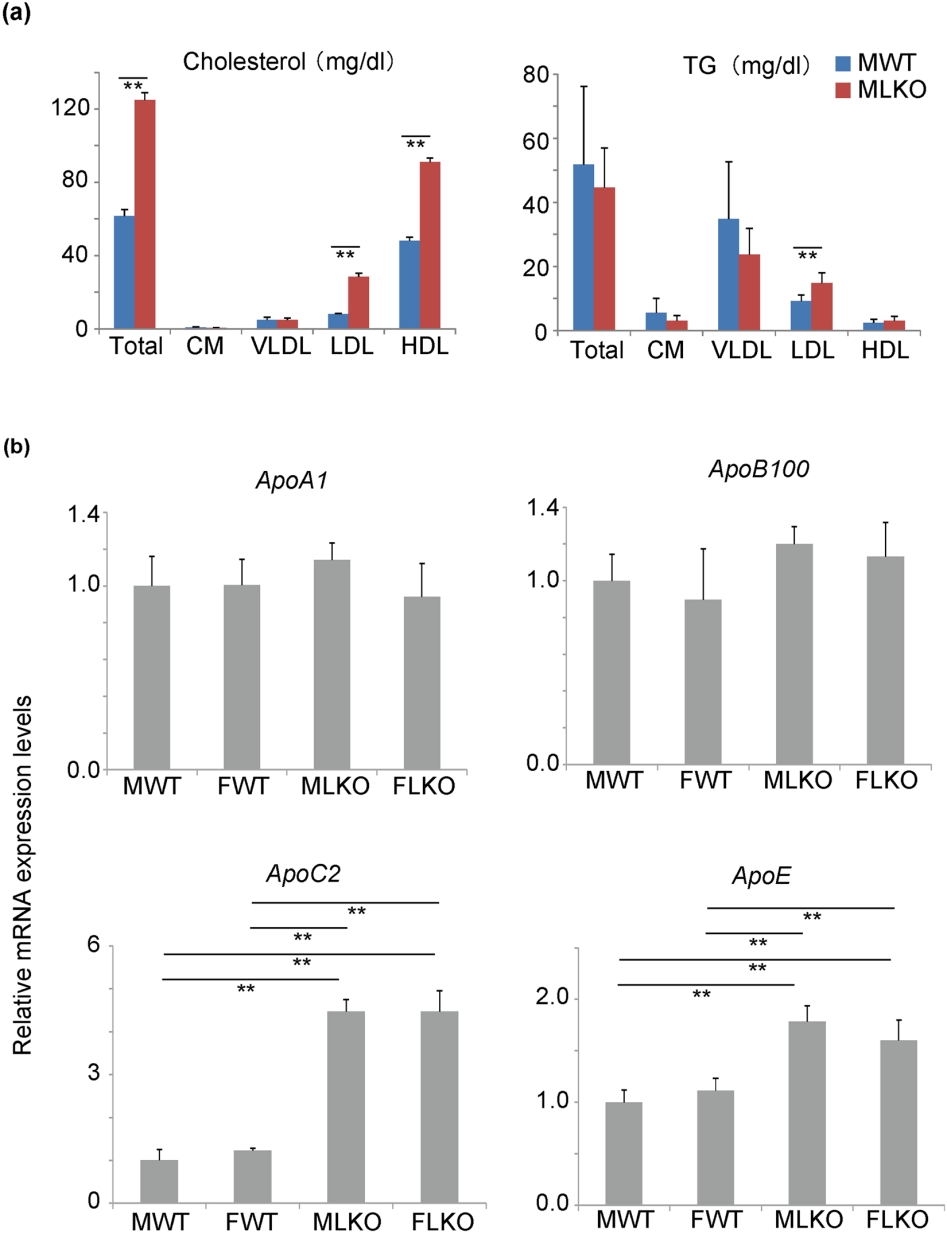
Analyses of lipoprotein transport in Bcl6-LKO mice (**a**) High-performance liquid chromatography analysis of serum cholesterol and triglyceride (TG) in 4 fractions of lipoproteins in mice fed with standard diet. Results are represented as mean ± standard deviation (S.D.) (n = 5 for male wild-type and Bcl6-LKO mice). CM, chylomicron; VLDL, very low-density lipoprotein; LDL, low-density lipoprotein; HDL, high-density lipoprotein. (**b**) The mRNA expression levels of *ApoA1*, *ApoB100*, *ApoC2*, and *ApoE* in standard diet-fed mouse livers were measured by quantitative real-time polymerase chain reaction. The *Hprt* gene was used as an internal control. The expression of genes in male wild-type mouse livers was set to 1.0. Results are represented as mean ± S.D. (n = 6 for male wild-type mice, n = 5 for female wild-type mice, n = 6 for male Bcl6-LKO mice, n = 4 for female Bcl6-LKO mice). **P < 0.01 MWT, male wild-type mouse samples; FWT, female wild-type mouse samples; MLKO, male Bcl6-LKO mouse samples; FLKO, female Bcl6-LKO mouse samples.

The Bcl6-LKO mice exhibited increased serum levels of total cholesterol and HDL cholesterol (Fig. 1c) and decreased hepatic levels of triglyceride (Fig. 2a). We hypothesised that lipoprotein transport might be different between Bcl6-LKO and WT mice. To verify this hypothesis, the serum lipoprotein profiles were analysed. Lipoproteins containing cholesterol and triglyceride can be fractionated based on size into chylomicron, very low-density lipoprotein (VLDL), low-density lipoprotein (LDL), and HDL. The levels of cholesterol and triglyceride in these lipoprotein fractions were measured. The cholesterol concentrations in the chylomicron and VLDL fractions were comparable between WT and Bcl6-LKO mice (Fig. 3a). The cholesterol concentrations in the total lipoprotein, LDL, and HDL fractions in Bcl6-LKO mice were significantly higher than those in WT mice. The serum triglyceride concentration was not significantly affected in most of the fractions in Bcl6-LKO mice, except LDL fraction.

Next, we analysed the expression of genes involved in lipoprotein metabolism by qRT-PCR. The mRNA expression levels of *ApoC2* and *ApoE* in Bcl6-LKO mice were upregulated when compared with those in WT mice (Fig. 3b). During the metabolic conversion of VLDL to LDL, the triglycerides in VLDL are hydrolysed into glycerine and free fatty acids. The fatty acids are then transported to the peripheral tissues. Lipoprotein lipase (LPL), an enzyme that catalyses this triglyceride hydrolysis, is reported to be activated by APOC2^13^. The deletion of Bcl6 in liver may promote APOC2-mediated changes in the composition of lipoproteins, including VLDL-LDL composition.

### Suppression of NASH progression induced by short-term CDAHFD feeding in Bcl6-LKO mice

Next, we analysed the role of hepatocytic Bcl6 in NASH progression. Previous studies are reported to be used a classical methionine and choline-deficient diet to induce liver lipid accumulation. Choline and methionine are required for the production of VLDL, which is important for transporting lipid components from the liver into the blood. Thus, the deficiency of choline and methionine promotes the accumulation of lipids in the liver and contributes to the progression of NASH. However, the consumption of a classical methionine and choline-deficient diet leads to a significant weight loss, which is not suitable for the NASH pathological model^14^. Therefore, the mice were fed with CDAHFD, which lacks choline and is supplemented with 0.1 weight by weight (W/W) % methionine (Research diet, A06071302), in this study. This diet is reported to significantly induce hepatic lipid accumulation and hepatocytic injury without inducing weight loss^15^.

**Figure 4:**
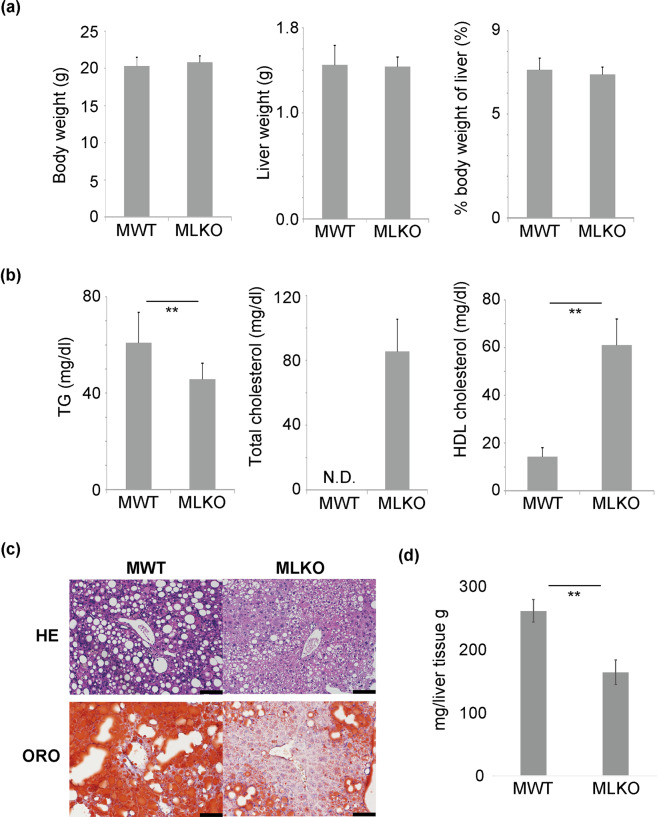
Analysis of characteristics of Bcl6-LKO mice fed with choline-deficient L-amino acid-define, high-fat diet (CDAHFD) for 7 weeks. (**a**) Body weight and liver weight were measured. The percent weight of liver relative to the body weight (% body weight of liver) was calculated. Results are represented as mean ± standard deviation (S.D.) (n = 9 for male wild-type mice, n = 10 for male Bcl6-LKO mice). (**b**) The serum levels of triglyceride (TG), total cholesterol, and high-density lipoprotein (HDL) cholesterol were measured. Results are represented as mean ± S.D. (n = 9 for male wild-type mice, n = 10 for male Bcl6-LKO mice). The levels of total serum cholesterol in all MWT samples were below the detection limits (50 mg/dL). Thus, the datapoint is shown as N.D. (Not detected). (**c**) Analyses of lipid accumulation in livers. The livers of WT and Bcl6-LKO mice fed with CDAHFD for 7 weeks were subjected to haematoxylin and eosin (HE) staining and oil red O (ORO) staining. Representative images are shown. Scale bar shows 100 μm. (**d**) The triglyceride levels in the livers of WT and Bcl6-LKO mice fed with CDAHFD were quantified. Results are represented as mean ± S.D. (n = 4). **P < 0.01 MWT, male wild-type mouse samples; MLKO; male Bcl6-LKO mouse samples.

The WT and Bcl6-LKO mice were fed with a standard diet until 6 age in weeks before being fed with CDAHFD. As mentioned above, the body weight of Bcl6-LKO mice aged 6 weeks was slightly lower than that of age-matched WT mice (Supplementary Fig. S1b). The intake of first 1 week CDAHFD by Bcl6-LKO mice was slightly lower than that of age-matched WT mice. However, there was no significant difference in the body weight and food intake between Bcl6-LKO and WT mice, when these mice were fed with CDAHFD for 2- to 7-weeks (Fig. 4a, Supplementary Fig. S1b and S1c). This result suggested that the phenotypic changes in Bcl6-LKO mice fed with CDAHFD were due to Bcl6 liver-deletion. The liver weight and percent weight of liver relative to the body weight were not significantly different between WT and Bcl6-LKO mice fed with total 7 week CDAHFD (Fig. 4a). The analysis of the serum lipid component revealed that the Bcl6-LKO mice exhibited decreased levels of serum triglyceride (Fig. 4b). The consumption of CDAHFD suppressed VLDL synthesis in the liver. Thus, the serum total cholesterol level in WT mice was significantly low. In contrast, the total cholesterol level was at the physiological level in Bcl6-LKO mice. The serum HDL cholesterol levels in Bcl6-LKO mice were higher than those in WT mice. Next, we analysed the hepatic lipid accumulation by oil red O staining (ORO). The accumulation of lipids in the liver markedly increased in WT mice after short-term CDAHFD feeding. However, the Bcl6-LKO mice exhibited decreased degree of steatosis after short-term CDAHFD feeding (Fig. 4c). The liver triglyceride level was significantly suppressed in Bcl6-LKO mice (Fig. 4d), which suggested that Bcl6 is important for lipid accumulation during NASH progression.

**Figure 5:**
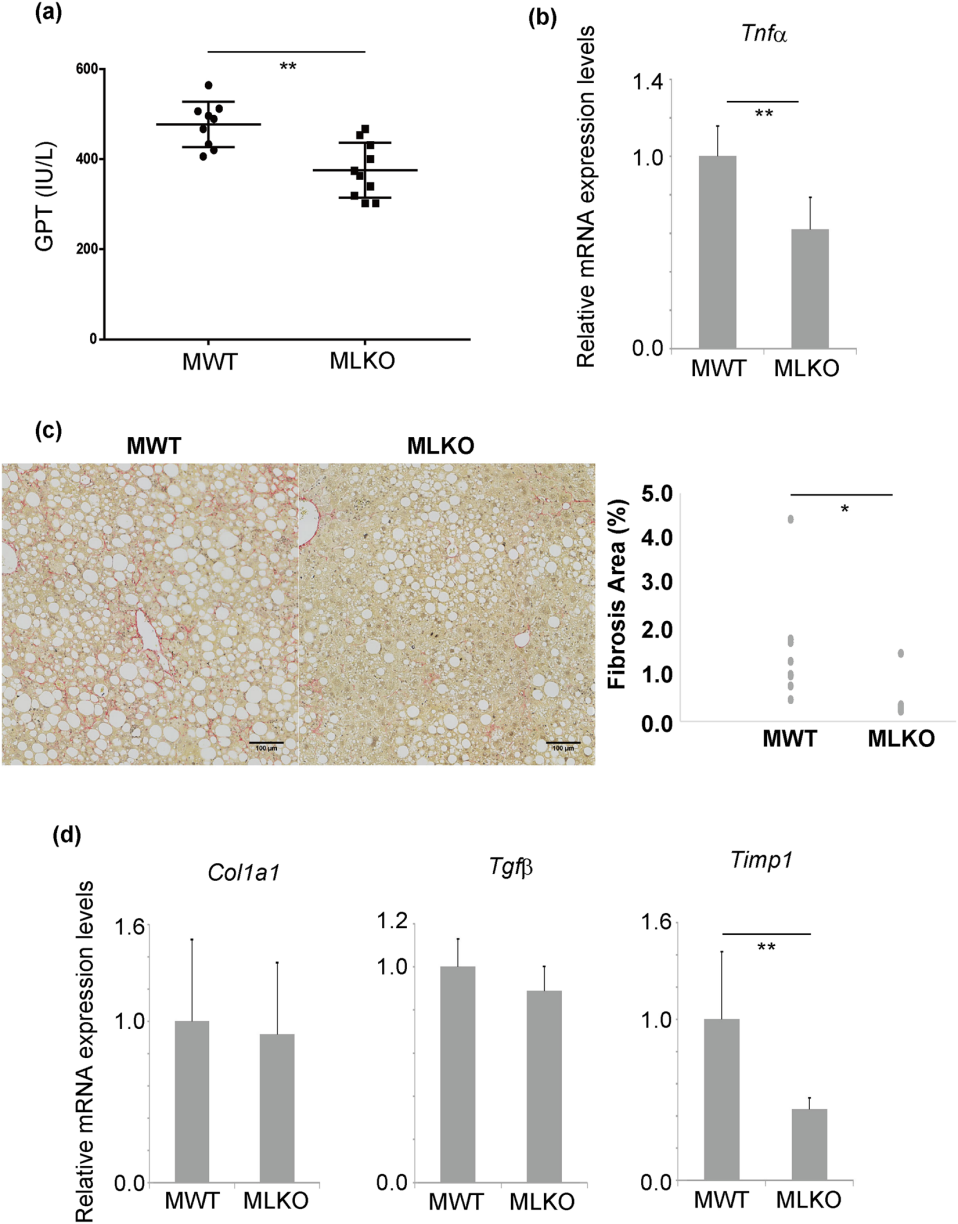
Non-alcoholic steatohepatitis (NASH) pathologies induced by short-term CDAHFD feeding in Bcl6-LKO mice (**a**) Assessment of liver injury. The serum level of glutamic pyruvic transaminase (GPT) was measured as an indicator of liver injury. Results are represented as mean ± standard deviation (S.D.) (n = 9 for male wild-type mice, n = 10 for male Bcl6-LKO mice). (**b**) The mRNA expression level of *Tnfα* in the liver was measured by quantitative real-time polymerase chain reaction (qRT-PCR) as an indicator of liver inflammation. The *Hprt* gene was used as an internal control. The expression of genes in male wild-type mouse livers was set to 1.0. Results are represented as mean ± S.D. (n = 4 for male wild-type mice, n = 7 for male Bcl6-LKO mice). (**c**) Liver fibrosis induced by short-term CDAHFD feeding. Sirius red stain was performed to visualise the liver fibres and quantify the amount of liver fibres using the ImageJ software. The scale bar shows 100 μm. Results are represented as mean ± S.D. (n = 9 for male wild-type mice, n = 10 for male Bcl6-LKO mice). (**d**) The mRNA expression levels of liver fibrosis markers were measured by qRT-PCR. The *Hprt* gene was used as an internal control. The expression of genes in male wild-type mouse livers was set to 1.0. Results are represented as mean ± S.D. (n = 9 for male wild-type mice, n = 10 for male Bcl6-LKO mice). *P < 0.05, **P < 0.01 MWT, male wild-type mouse samples; MLKO: male Bcl6-LKO mouse samples.

The progression of NASH is associated with hepatic damage, liver inflammation, and liver fibrosis^16,17^^,^. The GPT levels, which indicate the degree of liver injury, were significantly lower in Bcl6-LKO mice than in WT mice (Fig. 5a). The mRNA expression levels of tumour necrosis factor alpha (*Tnfα*) in Bcl6-LKO mice were significantly downregulated when compared with those in WT mice (Fig. 5b). The mRNA expression levels of *Il6* in Bcl6-LKO mice were significantly downregulated when compared with those in WT mice. In contrast, the expression levels of *Il1b* were similar between WT and Bcl6-LKO mice (Supplementary Fig. S3). Furthermore, liver fibrosis was assessed by Sirius red staining. The quantification of Sirius red-positive collagen fibres is shown in Fig. 5c. Liver fibrosis was significantly induced in WT male mice fed with CDAHFD. In contrast, the Bcl6-LKO mice exhibited suppressed accumulation of extracellular matrices. The mRNA expression levels of fibrosis markers, such as collagen 1a1 (*Col1a1)*, transforming growth factor beta (*Tgf β*), and tissue inhibitor of metalloproteinase 1 (*Timp1)* in the liver were measured by qRT-PCR. The mRNA expression levels of *Col1a1* and *Tgfβ* were similar between WT and Bcl6-LKO mice. However, the mRNA expression levels of *Timp1* were significantly downregulated in Bcl6-LKO mice (Fig. 5d). These results suggest that mice fed with CDAHFD for 7 weeks exhibited hepatic lipid accumulation, hepatic inflammation, and mild fibrosis. These NASH-like pathological changes induced by short-term CDAHFD feeding were attenuated in Bcl6-LKO mice. Additionally, the expression levels of genes related to β-oxidation (*Abcd1*, *Acnat2*, *Acot2*, *Acot4*, enoyl-CoA hydratase and 3-hydroxyacyl CoA dehydrogenase (*Ehhadh*), *Hadh*, *Acadvl*, and *Ucp2*) were analysed in NASH model mouse livers (Supplementary Fig. S4). The expression levels of several β-oxidation genes were upregulated in the livers of Bcl6-LKO mice even after the induction of NASH.

### Suppression of liver tumours induced by long-term CDAHFD feeding in Bcl6-LKO mice

**Figure 6:**
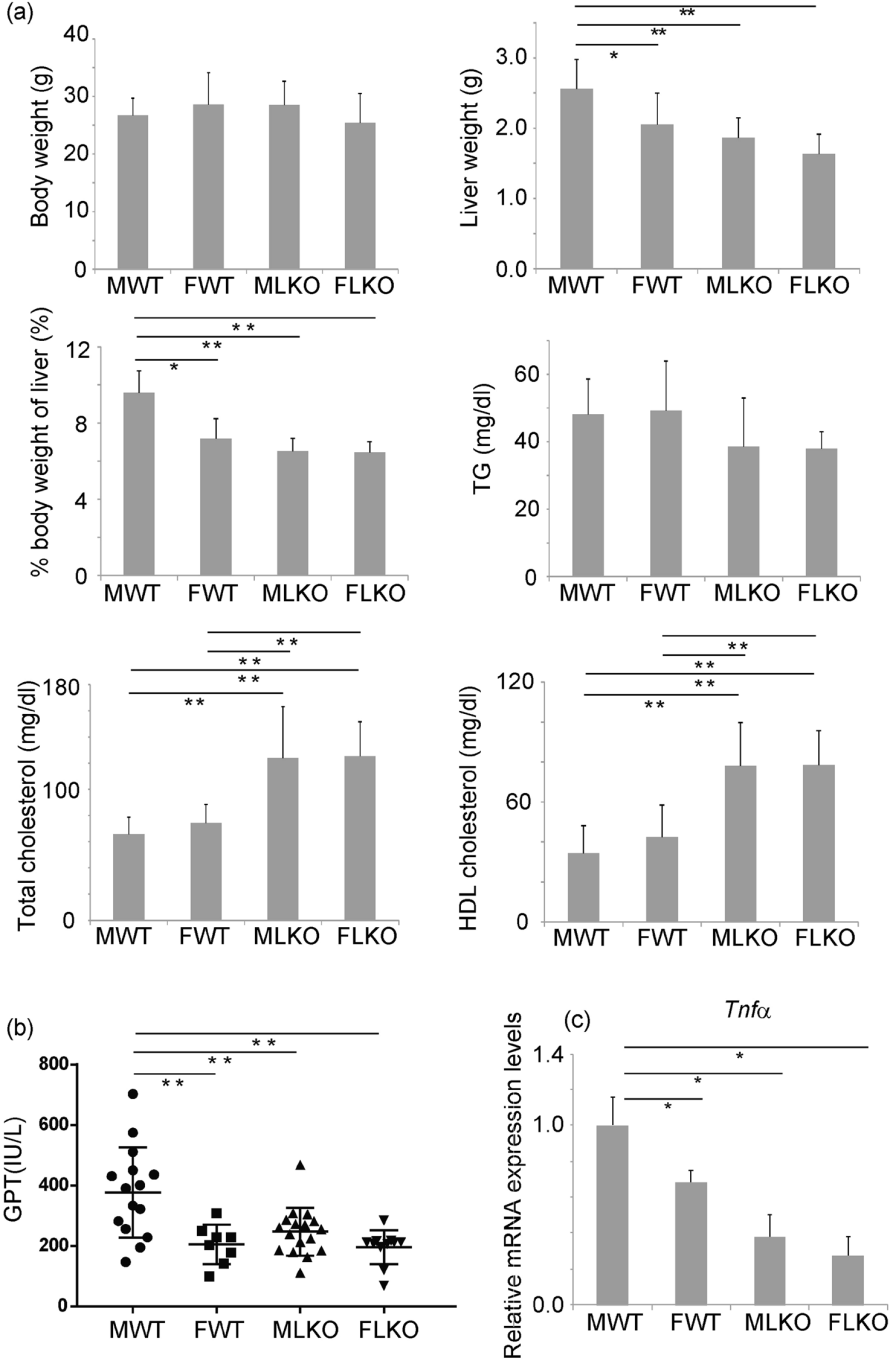
Analysis of characteristics of Bcl6-LKO mice fed with CDAHFD for 38 weeks. (**a**) Body weight and liver weight were measured. The percent weight of liver relative to the body (% body weight of liver) weight was calculated. The serum levels of triglyceride (TG), total cholesterol, and high-density lipoprotein (HDL) cholesterol levels were measured. Results are represented as mean ± standard deviation (S.D.) (n = 15 for male wild-type mice, n = 8 for female wild-type mice, n = 17 for male Bcl6-LKO mice, n = 11 for female Bcl6-LKO mice). (**b**) The serum levels of glutamic pyruvic transaminase (GPT) were measured as an indicator of liver injury. Results are represented as mean ± S.D. (n = 15 for male wild-type mice, n = 8 for female wild-type mice, n = 17 for male Bcl6-LKO mice, n = 11 for female Bcl6-LKO mice). (**c**) The mRNA expression levels of *Tnfα* mRNA in the liver were measured by quantitative real-time polymerase chain reaction. The *Hprt* gene was used as an internal control. The expression of genes in male wild-type mouse livers was set to 1.0. (n = 10 for male wild-type mice, n = 6 for female wild-type mice, n = 10 for male Bcl6-LKO mice, n = 10 for female Bcl6-LKO mice). *P < 0.05, **P < 0.01. MWT, male wild-type mouse samples; FWT, female wild-type mouse samples; MLKO, male Bcl6-LKO mouse samples; FLKO, female Bcl6-LKO mouse samples.

Next, we analysed the role of Bcl6 in the pathogenesis of NASH-derived liver tumours. The long-term CDAHFD feeding is reported to induce liver tumours^18^. In this study, the Bcl6-LKO mice were fed with CDAHFD for 38 weeks to induce liver tumours. The consumption of CDAHFD for 38 weeks did not affect the body weight of either WT or Bcl6-LKO mice. The percent weight of liver relative to the body weight in male WT mice was significantly increased when compared with that in WT female and Bcl6-LKO mice. Similar to short-term CDAHFD feeding, the serum cholesterol levels (both total and HDL cholesterol) in Bcl6-LKO mice were significantly higher than those in WT mice after 38 weeks of CDAHFD feeding (Fig. 6a).

The GPT level in WT female mice was significantly lower than that in WT male mice (Fig. 6b). The mRNA expression levels of *Tnfα* were significantly downregulated in WT female (Fig. 6c). These results suggest that CDAHFD-induced liver injury and inflammation vary depending on the gender. Furthermore, both the GPT level and *Tnfα* mRNA expression levels in Bcl6-LKO mice were significantly lower than those in WT male mice.

**Figure 7:**
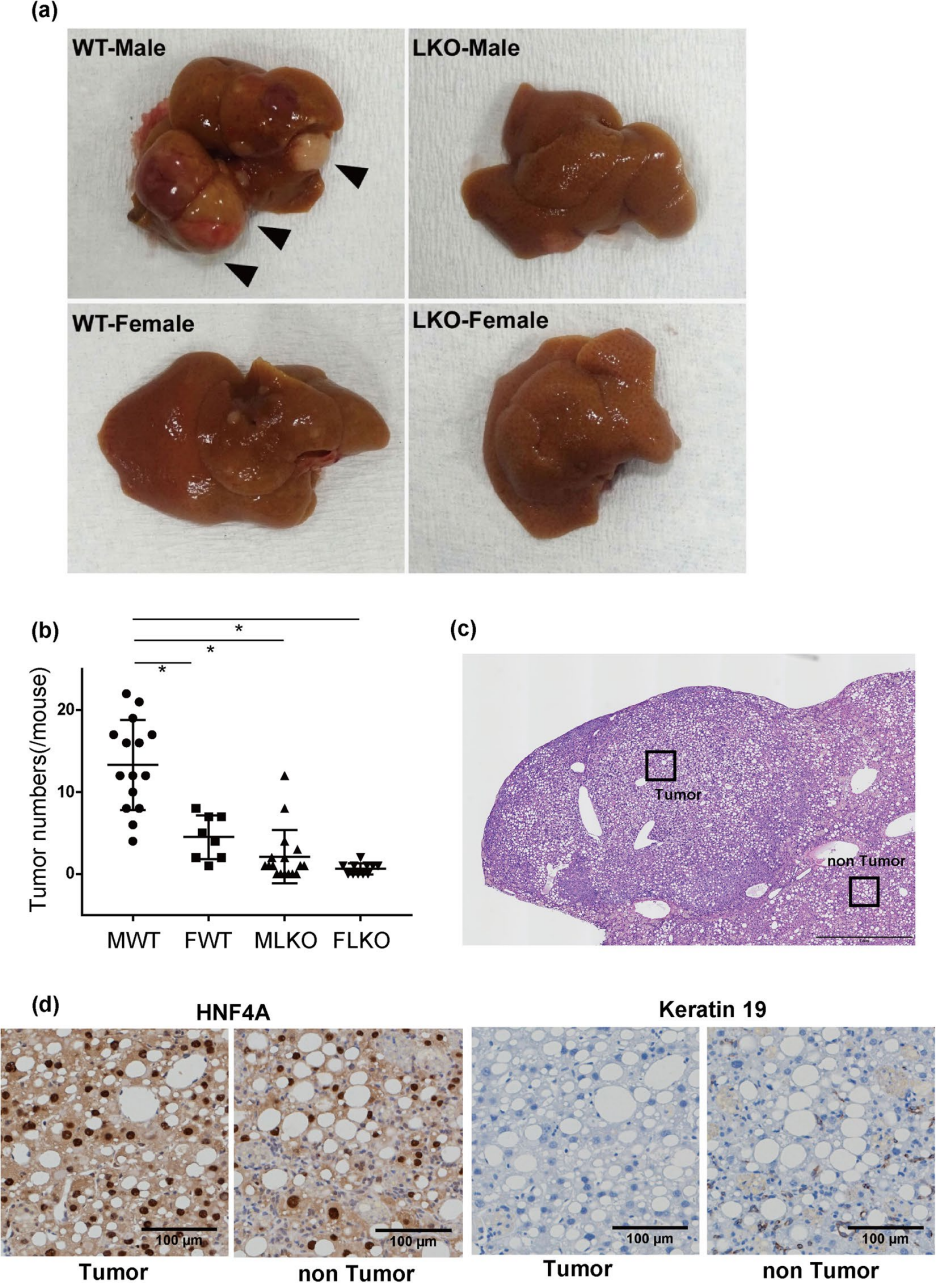
NASH-induced liver tumours in Bcl6-LKO mice (**a**) WT and Bcl6-LKO mice were fed with CDAHFD for 38 weeks. Macro images of liver tumours induced are shown. (**b**) The number of liver tumours induced by 38 weeks CDAHFD feeding was counted. Results are represented as mean ± standard deviation (S.D.) (n = 15 for male wild-type mice, n = 8 for female wild-type mice, n = 17 for male Bcl6-LKO mice, n = 11 for female Bcl6-LKO mice). *P < 0.05. MWT, male wild-type mouse samples; FWT, female wild-type mouse samples; MLKO, male Bcl6-LKO mouse samples; FLKO, female Bcl6-LKO mouse samples. (**c,d**) Analysis of liver tumours induced by long-term CDAHFD feeding. Haematoxylin and eosin staining and immunohistochemical staining were performed. Representative images are shown. The scale bar shows 1 mm in (**c**) and 100 μm in (**d**).

Severe NASH was reported to induce hepatocarcinogenesis in humans^19,20^^,^^21^^,^. In this study, long-term CDAHFD feeding (38 weeks) induced the formation of tumours in the liver. The number of liver tumours with a diameter of more than 1 mm was lesser in male Bcl6-LKO mice (average 2.1/mouse) than that in WT male mice (average 13.3/mouse). Only few numbers of liver tumours were detected in female WT (average 4.5/mouse) or female Bcl6-LKO mice (average 0.6/mouse) (Fig. 7a,b). The histopathological analyses revealed that the tumours comprised tumour cells exhibiting nuclear HNF4α expression. This indicated that these tumour cells were well-differentiated (Fig. 7c,d). The expression of keratin (KRT) 19 was not detected in the tumour cells and steatotic hepatocytes. The expression of KRT19 was detected in bile ductules scattered in the non-tumorous area (ductular reaction).

## Discussion

This study demonstrated that the liver-specific knockout of Bcl6 affected the serum cholesterol and liver triglyceride levels in mice. The short-term CDAHFD feeding resulted in the induction of NASH-like pathologies, such as liver injury, hepatic lipid accumulation, and liver fibrosis in WT mice, which were suppressed in Bcl6-LKO mice. Moreover, long-term CDAHFD feeding-induced liver tumours in WT mice, which was specific to male gender, was suppressed in Bcl6-LKO mice. These results suggest that Bcl6 is involved in the pathogenesis of NASH and NASH-derived tumours.

The function of Bcl6 in fatty acid metabolism has been analysed using whole-body Bcl6 knockout mice^9^. In addition to hepatic fatty acid accumulation, the whole-body Bcl6 knockout mice exhibit decreased body weight and liver weight caused due to atrophy of adipose tissue. The deletion of Bcl6 downregulated the expression of *Chrebp*, which subsequently downregulates the expression of lipid synthesis enzyme *Fasn* and lipid modification enzyme *Scd1*^9^. This is because CHREBP is the transcriptional factor regulating the expression of these genes^22^. The fatty acid synthesis system is downregulated in the whole-body Bcl6 knockout mouse, which leads to decreased fatty acid levels in the liver and body. Similar to whole-body Bcl6 KO mice, the Bcl6 LKO mice exhibited decreased triglyceride accumulation in the liver. In contrast, the mRNA expression levels of *Chrebp* and *Fasn* were similar between WT and Bcl6 LKO mice in this study. These results indicate that hepatocytic Bcl6 regulates fatty acid accumulation in the liver through a mechanism that is different from the Fasn-dependent lipid synthesis pathways. It was suggested that Bcl6 expression in non-liver tissues, such as adipose tissues, can promote lipid metabolic changes in whole-body Bcl6 knockout mice. Previously, we performed microarray expression analysis using Bcl6-LKO mouse livers (shown in GSE89091 and GSE107435)^11^, which revealed changes in the expression of several fatty acid-modifying enzymes. These lipid modification genes may affect fatty acid metabolism in the Bcl6-deficient liver.

NASH is induced by excessive accumulation of fatty acids in the liver. In mice, CDAHFD feeding can induce excess fatty acid accumulation and liver injury by inhibiting VLDL synthesis. The Bcl6-LKO mice exhibited suppressed accumulation of fatty acids and suppressed level of liver injury and liver fibrosis, which were induced by CDAHFD feeding. Bcl6 in the liver is previously reported to be involved in the expression of β-oxidation-related genes of fatty acids^12^. Our study revealed that the expression of β-oxidation-related genes was upregulated in Bcl6-LKO mice even after induction of NASH. The fatty acids accumulated in the liver induced by VLDL synthesis inhibition may undergo β-oxidation. Interestingly, the Bcl6-LKO mice fed with CDAHFD exhibited physiological levels of total and HDL cholesterol, which suggest that Bcl6 regulates the liver cholesterol metabolic pathways during NASH progression.

The liver tumours can be induced with high efficiency during NASH progression by long-term CDAHFD feeding^18^. In this study, NASH-induced liver tumours, which were observed in male mice, were dependent on Bcl6. Recently, we had reported about the Bcl6-mediated regulation of liver functional genes, especially gender-specific drug metabolic genes^11^. These results suggest a correlation between gender and progression of liver diseases and tumours. Additionally, NASH progression in both human and mouse is reported to be related to gender and is regulated by menopause^23,24^^,^. It is suggested that sex hormones, such as oestrogen exerted a protective effect on liver diseases. In diethylnitrosamine (DEN)-induced hepatocellular carcinoma mouse models, male-specific production of IL6 was reported to induce hepatocellular carcinoma. In female mice, oestrogen inhibited the DEN-induced production of IL6 and the development of hepatocellular carcinoma^25^. In our study, the expression levels of *Il6* in Bcl6-LKO mice were downregulated when compared with those in WT mice (Supplementary Fig. S3). In contrast, the serum levels of β-oestradiol, the most active oestrogen, in male WT and male Bcl6-LKO mice were comparable^11^. Liver-specific Bcl6-deficient mice have lost the gender differences in expression of several liver function genes such as drug-metabolizing enzymes, and livers of male Bcl6-LKO mice show expression patterns similar to female WT mice due to hepatocytic Bcl6-deficiency^11^. The fertility and blood sex hormone levels were not affected in Bcl6-LKO mice, indicating that Bcl6 specifically changed liver gender-dependent functions. These results suggested that the signalling pathway repressing NASH-induced liver tumours in male Bcl6-LKO mice is regulated by the repression of IL6 production and Bcl6-related mechanisms regulating gender-different liver functional genes. There is a possibility that liver tumours did not develop in wild-type female mice or liver-specific Bcl6-deficient mice because these mouse livers have female-type gene expression patterns of liver functional genes. Additionally, Bcl6 is reported to be an oncogene that regulates lymphoma progression^8^. We have the hypothesis that Bcl6 might also directly function as an oncogene during NASH-induced hepatocarcinogenesis. Future studies must focus on the interaction between Bcl6-downstream signals and liver tumour progression.

NASH progression and NASH-derived tumours were suppressed in the Bcl6-LKO mice. Therefore, Bcl6 can be a potential therapeutic target for NASH and NASH-induced liver tumours. Several low-molecular-weight compounds inhibiting Bcl6 functions have been developed as therapeutic agents for lymphomas^26,27^^,^^28^^,^^29^^,^. These molecules inhibit the interactions between Bcl6 and its coupling factors (NCoR, SMRT, and BCoR) in the haematopoietic cells. These inhibitors might be useful for the regulation of Bcl6-induced hepatic lipid accumulation. Additionally, Bcl6 can interact with other molecules and regulate gene expression. The analyses of Bcl6-interacting molecules and Bcl6-downstream targets in normal and lipid-accumulated livers are important to improve our understanding of NASH pathology and therapies.

## Methods

### Mice and animal experiments

Albumin promoter-Cre transgenic mice were from Jackson Laboratory (Bar Harbor, ME)^30,31^^,^. Bcl6-floxed mice^32^ were procured from RIKEN BRC (Tsukuba, Japan). The Bcl6-LKO mice were generated by mating the albumin promoter-Cre mice with Bcl6-floxed mice. The Cre-negative mice with the floxed alleles were used as the Bcl6-WT mice. The Cre-positive mice with the Bcl6-floxed alleles were used as Bcl6-LKO mice^11^. These mice were fed with a standard diet. To induce liver lipid accumulation, 6-week-old mice were fed with CDAHFD (A06071302, Research Diets, New Brunswick, NJ, USA), which completely lacks choline and is supplemented with 0.1 W/W % methionine, for 7 (short-term stimulation) or 38 weeks (long-term stimulation). For measuring the time course of changes in body weight or food intake, the mice were raised in an individual cage. The body weight and food intake were measured once or twice in a week. All animal experiments were performed in accordance with the approved guidelines. The animal experimental protocols were approved by the Institutional Animal Care and Use Committee at Tokai University (permit number: 193033).

### Serum analyses

The mice fed with standard diet or CDAHFD were dissected. The blood sample was collected from the heart. The serum was separated from the blood using Bloodsepar (Immuno-Biological Laboratories Co., Ltd, Gunma, Japan). The serum levels of total cholesterol, HDL cholesterol, triglyceride, and GPT were measured by Spotchem (Arkray. Inc, Kyoto, Japan). The range of measurements of each parameter is as follows: total cholesterol, 50–400 mg/dL; HDL cholesterol, 10–150 mg/dL; triglyceride, 25–500 mg/dL; GPT, 10–1000 IU/L. The samples in which the levels of parameters that were lower than the detection limits were excluded from the analyses, except for GPT. When the GPT level was lower than the detection limits, it was considered as 10 IU/L, which was the lowest detection limit.

### Liver triglyceride measurement

The livers were frozen in liquid nitrogen immediately after dissection and were stored at −80 °C until analysis. The liver triglyceride levels in mice fed with standard diet were analysed using the Triglyceride colorimetric assay kit (Cayman chemical, Ann Arbor, MI, USA). The liver triglyceride levels in mice fed with CDAHFD for 7 weeks were analysed using FOLCH method^33^ in SkylightBiotech (Akita, Japan).

### Serum lipid analyses in lipoproteins by gel filtration high-performance liquid chromatography (HPLC)

The blood sample was collected from the heart. The serum was separated from the blood using Bloodsepar. The serum was subjected to gel filtration HPLC in Skylight Biotech. The concentrations of triglyceride and cholesterol in each lipoprotein fraction were measured.

### Histological analysis

The liver samples were fixed in 4% paraformaldehyde (PFA) overnight and embedded in paraffin. The paraffin-embedded sections were subjected to haematoxylin and eosin staining and Sirius red staining using standard protocols^34,35^. To analyse the hepatic accumulation of lipids, the livers fixed in 4% PFA were embedded in optimum cutting temperature compound (Sakura Finetek Japan, Co., Ltd. Tokyo, Japan). These cryosections were subjected to ORO staining^36^. The images of the pathological specimens were captured using a BX63 microscope (Olympus, Tokyo, Japan). The area of Sirius red-positive fibres relative to the total area was calculated using ImageJ. In these analyses, large veins in the sections were eliminated.

The tumour tissues were subjected to HNF4 α and KRT19 immunohistochemical staining. The paraffin-embedded sections were heated at 110 °C for 10 min in a target retrieval solution (Dako, Santa Clara, CA, USA). The sections were blocked and then incubated with anti-HNF4α antibody (sc-6556, Santa Cruz Biotechnology, Dallas, TX, USA) or anti-KRT 19 antibody (provided by Dr. Miyajima) overnight at 4 °C. The sections were washed and incubated with Simple Stain horseradish peroxidase (HRP)-conjugated anti-goat IgG and anti-rabbit IgG antibodies (Nichirei Biosciences Inc., Tokyo, Japan) for 20 min at room temperature. The sections were visualised using diaminobenzidine (DAB) substrate and haematoxylin counter staining. The images of the specimens were captured under a BX63 microscope.

### Detection of mRNA by qRT-PCR

The liver samples stored in RNAlater solution (Thermo Fisher Scientific, Waltham, MA, USA) at −80 °C were used for mRNA expression analysis. Total RNA was extracted using TRIzol RNA Isolation Reagents (Thermo Fisher Scientific), following the manufacturer’s instructions. First-strand cDNA was synthesised from the extracted RNA for qRT-PCR analysis using the ReverTra Ace qPCR RT Master Mix with genome remover (TOYOBO, Osaka, Japan). The expression of target genes was normalised to that of hypoxanthine guanine phosphoribosyl transferase (*Hprt*). Quantitative analyses of the target mRNAs were performed using the Universal Probe Library System (Roche Diagnostics, Basal, Switzerland). The primers and probes used for qRT-PCR analysis are shown in Table 1.

**Table 1:** qRT-PCR primers for detection of mouse gene expression.

Mouse gene	Forward primer (5′→3′)	Reverse primer (5′→3′)	Probe number
Hprt	tcctcctcagaccgctttt	cctggttcatcatcgctaatc	95
Fasn	gctgctgttggaagtcagc	agtgttcgttcctcggagtg	58
Scd1	ttccctcctgcaagctctac	cagagcgctggtcatgtagt	34
Chrebp	ggcctggctggaacagta	cgaagggaattcaggacagt	108
ApoA1	gcggcagagactatgtgtcc	cagttttccaggagattcaggt	63
ApoB100	tccagacaacctcttcctaaagac	ggatgtcaatgtttattttgttcct	53
ApoC2	cccttcctgccactacattc	caacatcaggatgaccagga	72
ApoE	agaccctggaggctaaggac	agagccttcatcttcgcaat	12
Tnfα	tcttctcattcctgcttgtgg	ggtctgggccatagaactga	49
Col1a1	acctaagggtaccgctgga	tccagcttctccatctttgc	19
Tgfβ	tggagcaacatgtggaactc	gtcagcagccggttacca	72
Timp1	gcaaagagctttctcaaagacc	agggatagataaacagggaaacact	76
Abcd1	gttctaccacaggcccaagt	catcaatgctcacggcacta	79
Acnat2	gagcaaggaaaacatacagtctca	aactggatcatcatcaaggtgtt	69
Acot2	ccccaagagcatagaaacca	ccaattccaggtccttttacc	83
Acot4	atgcttcgacatccaaaggt	ggaagccatgatcagacagac	17
Ehhadh	ccggtcaatgccatcagt	ctaaccgtatggtccaaactagc	109
Hadh	tggatactacaaagttcatcttgga	aaggactgggctgaaataagg	106
Acadvl	ggtggtttgggcctctcta	gggtaacgctaacaccaagg	53
Ucp2	acagccttctgcactcctg	ggctgggagacgaaacact	2
Il6	gctaccaaactggatataatcagga	ccaggtagctatggtactccagaa	6
Il1β	agttgacggaccccaaaag	agctggatgctctcatcagg	38

### Statistical analysis

Microsoft Excel 2010 (Microsoft, Redmond, WA, USA) was used to calculate the standard deviation (S.D.). The differences between different groups were analysed by Student’s two-tailed *t*-test. The differences between multiple groups were analysed by analysis of variance (ANOVA), followed by Turkey’s test in Prism7 (GraphPad Software, SD, USA).

## Supplementary information

Supplementary Information.

## Data Availability

No datasets were generated or analysed during the current study.
