# Supplementary material for: Liver-specific knockout of B cell lymphoma 6 suppresses progression of non-alcoholic steatohepatitis in mice
Source: Sci Rep. 2020 Jun 16;10:9704. doi: 10.1038/s41598-020-66539-z (PMC7297717; doi:10.1038/s41598-020-66539-z)
Supplement: Supplementary file 1 — Supplementary Information. [file 41598_2020_66539_MOESM1_ESM.docx]

**Liver-specific knockout of B cell lymphoma 6 suppresses progression of non-alcoholic steatohepatitis in mice**

**Hiromi Chikada^1,2^, Kinuyo Ida^1, 2^, Yuji Nishikawa^4^, Yutaka Inagaki^2, 3^, and Akihide Kamiya^1*^**

^1^Department of Molecular Life Sciences, Tokai University School of Medicine, 143 Shimokasuya, Isehara, Kanagawa, Japan 259-1193

^2^Center for Matrix Biology and Medicine, Tokai University School of Medicine, 143 Shimokasuya, Isehara, Kanagawa, Japan 259-1193

^3^Department of Innovative Medical Science, Tokai University School of Medicine, 143 Shimokasuya, Isehara, Kanagawa, Japan 259-1193

^4^Department of Pathology, Asahikawa Medical University, Asahikawa, Hokkaido, Japan.

***Correspondence:** Akihide Kamiya, Ph.D.

Department of Molecular Life Sciences, Tokai University School of Medicine, 143 Shimokasuya, Isehara, Kanagawa, Japan 259-1193, Telephone: +81-463-93-1121 ex 2783, FAX: +81-463-95-3522, E-mail: [kamiyaa@tokai-u.jp](mailto:kamiyaa@tokai-u.jp)

**Supplementary Figures S1, S2, S3, and S4**

**
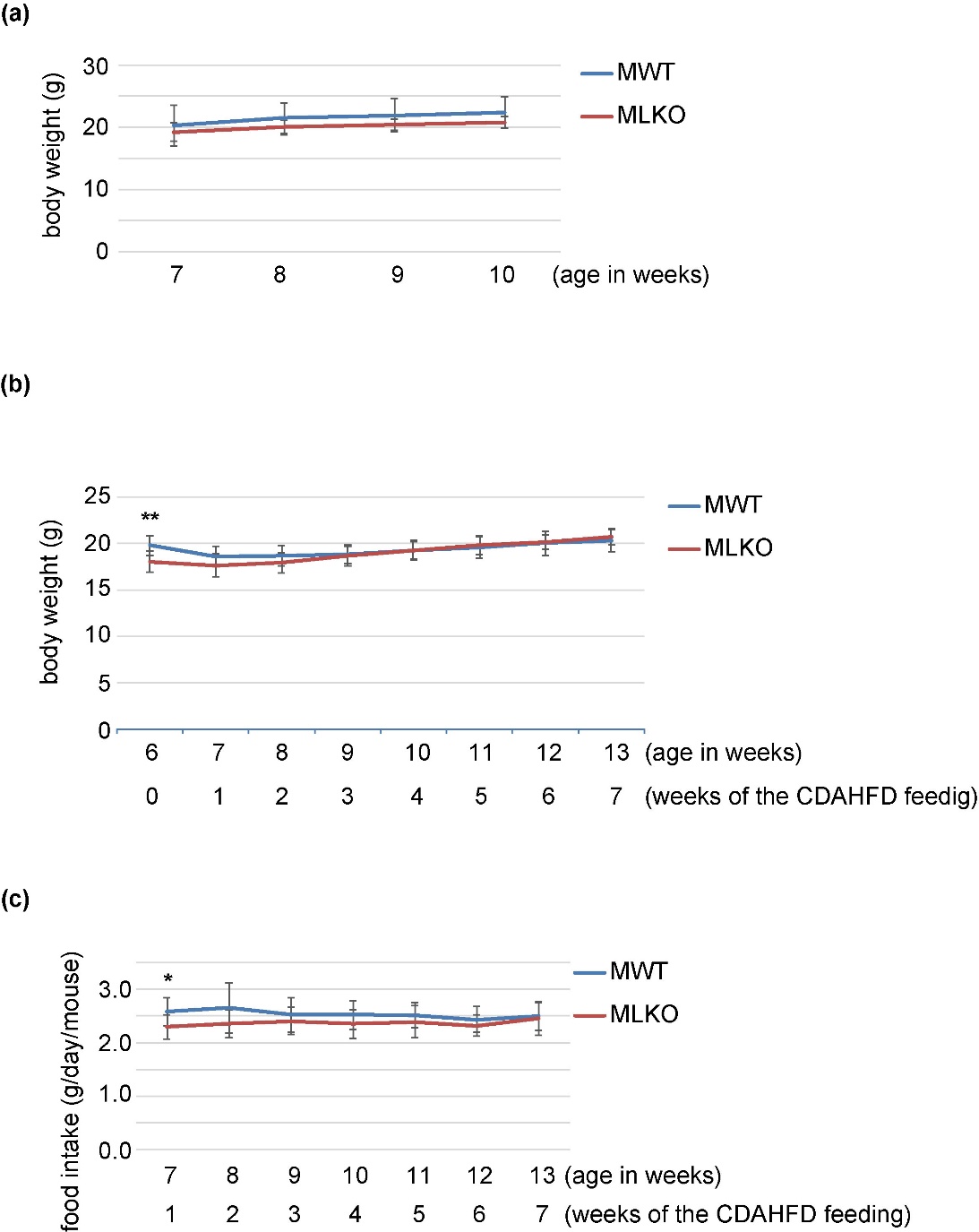
**

**Supplementary Figure S1** Time course of changes in body weight and food intake in standard diet feeding and CDAHFD feeding. (a) Body weight of mice aged 7-10 weeks in standard diet feeding was measured. Results are represented as mean ± standard deviation (S.D.) (n=6). (b) Body weight and (c) food intake were measured from the point when 6-week-old mice were first fed with choline-deficient, L-amino acid-defined, high-fat diet (CDAHFD). Results are represented as mean ± S.D. (n=9 for male wile-type mice, n=10 for male Bcl6-LKO mice). *P<0.05. **P<0.01. MWT, male wild-type mouse samples; MLKO, male Bcl6-LKO mouse samples.


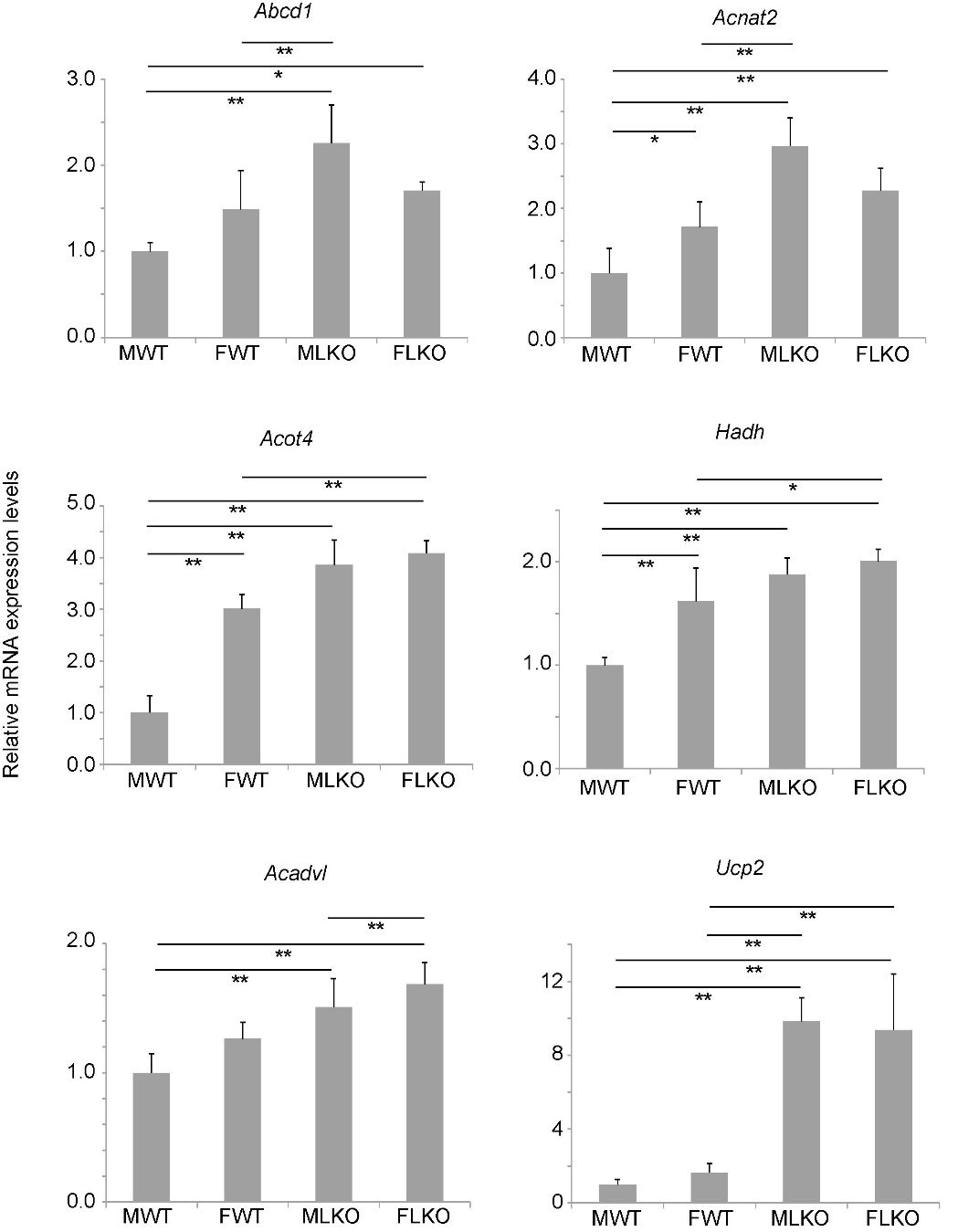


**Supplementary Figure S2** mRNA expression levels of genes related to fatty acid oxidation in mice fed with standard diet were measured by quantitative real-time polymerase chain reaction. The expression of genes in male wild-type mouse livers was set to 1.0. Results are represented as mean ± standard deviation (S.D.) (n=6 for male wild-type mice, n=5 for female wild-type mice, n=6 for male Bcl6-LKO mice, n=4 for female Bcl6-LKO mice). *P<0.05. **P<0.01. MWT, male wild-type mouse samples; FWT, female wild-type mouse samples; MLKO, male Bcl6-LKO mouse samples; FLKO, female Bcl6-LKO mouse samples.

**
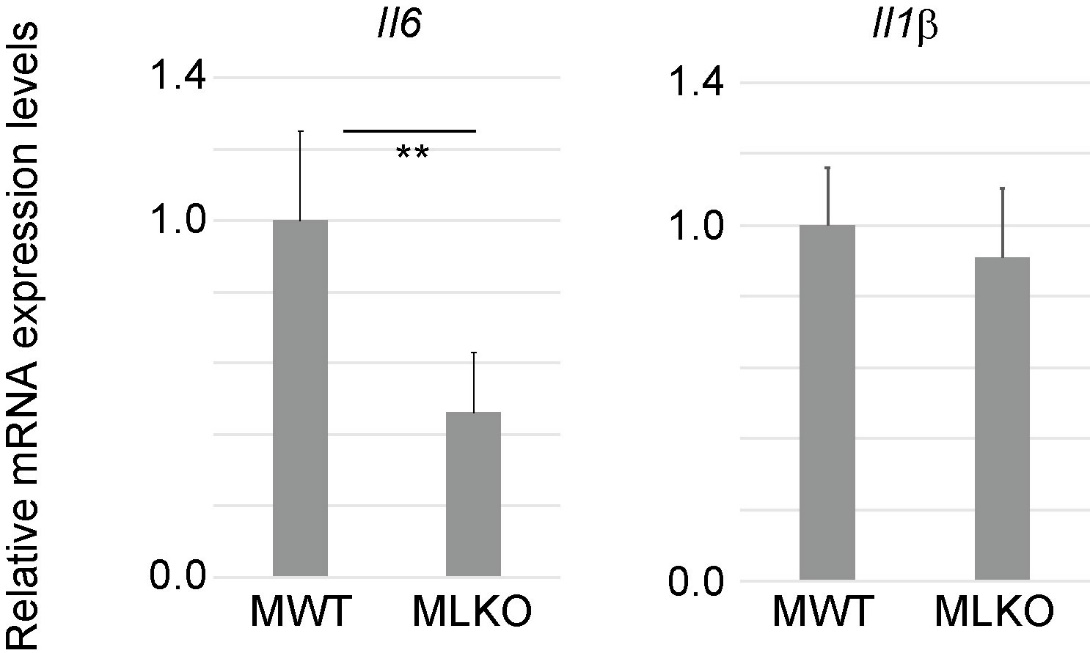
**

**Supplementary Figure S3** mRNA expression levels of *Il6* and *Il1β* in mice fed with CDAHFD for 7 weeks were measured by quantitative real-time polymerase chain reaction. The expression of genes in male wild-type mouse livers was set to 1.0. Results are represented as mean ± standard deviation (S.D.) (n=4 for male wild-type mice, m=7 for male Bcl6-LKO mice). **P<0.01. MWT, male wild-type mouse samples; MLKO, male Bcl6-LKO mouse samples.

**
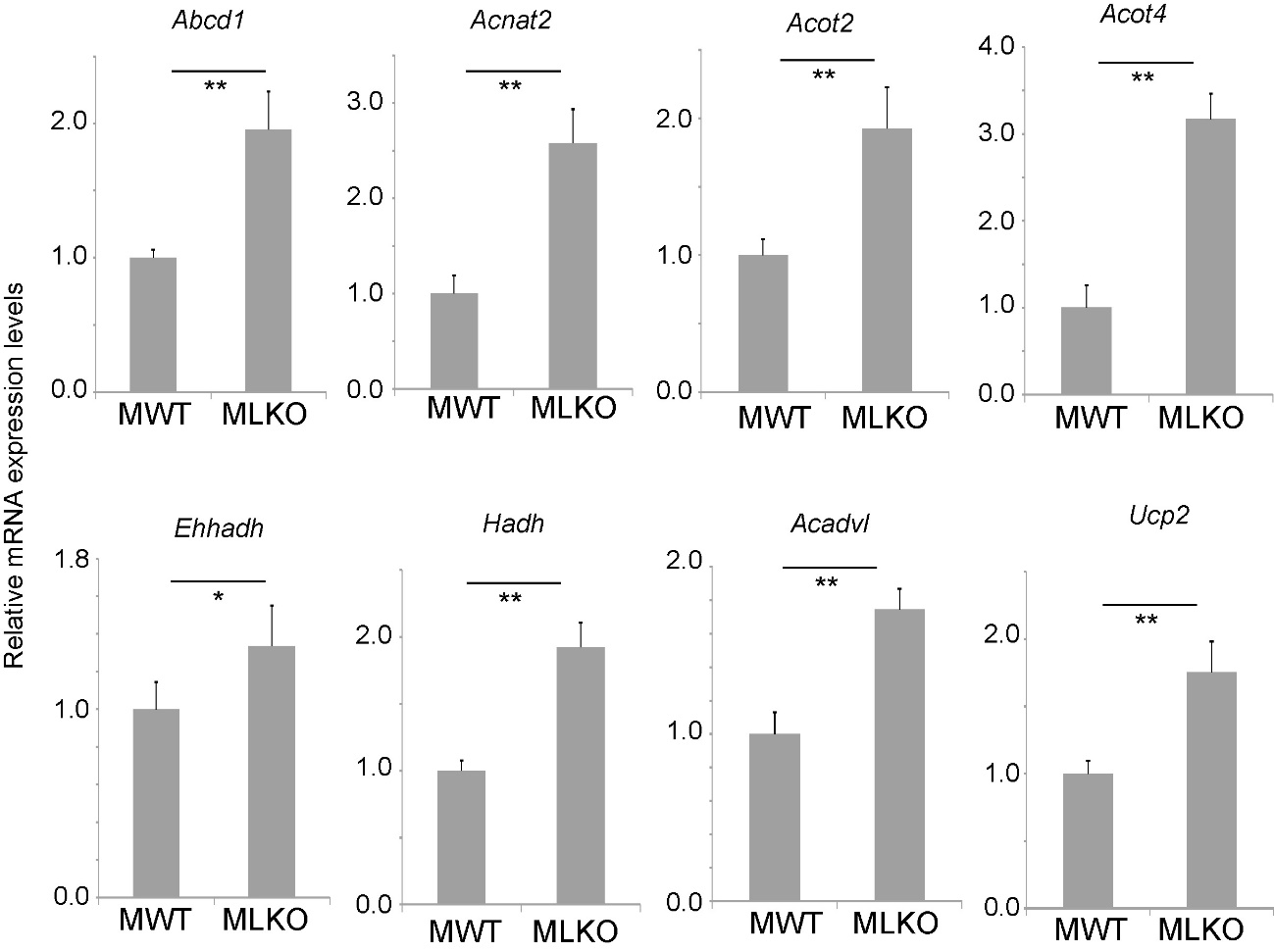
**

**Supplementary Figure S4** mRNA expression levels of genes related to fatty acid oxidation in mice fed with CDAHFD for 7 weeks was measured by quantitative real-time polymerase chain reaction. The expression of genes in male wild-type mouse livers was set to 1.0. Results are represented as mean ± standard deviation (S.D.) (n=4 for male wild-type mice, n=7 for male Bcl6-LKO mice). *P<0.05. **P<0.01. MWT, male wild-type mouse samples; MLKO, male Bcl6-LKO mouse samples.
